# Systemic Lupus Erythematosus Induced Central Variant of Posterior Reversible Encephalopathy Syndrome: A Rare Association

**DOI:** 10.7759/cureus.13431

**Published:** 2021-02-18

**Authors:** Gautam Jesrani, Samiksha Gupta, Yajur Arya, Arshi Syal, Monica Gupta

**Affiliations:** 1 Internal Medicine, Government Medical College and Hospital, Chandigarh, IND

**Keywords:** systemic lupus erythematosus, posterior reversible encephalopathy syndrome, magnetic resonance imaging

## Abstract

Involvement of the posterior cerebral structures is well established in patients with posterior reversible encephalopathy syndrome (PRES). In striking contrast to this, a subset of patients may have an atypical presentation characterized by the involvement of central structures. This is usually seen in the backdrop of systemic lupus erythematosus (SLE), a rare but known cause of PRES. Timely diagnosis with judicious use of radiological modalities and therapeutic intervention is key for successful recovery; however, the prognosis of PRES in patients with SLE is not very promising. We are describing an extremely rare case of a patient diagnosed with a central variant of PRES with previously undiagnosed SLE.

## Introduction

Posterior reversible encephalopathy syndrome (PRES) is a clinico-radiological diagnosis of cerebral edema syndrome, chiefly involving the posterior structures including parieto-occipital, posterior fronto-cortical and subcortical areas of the brain. PRES may result from a wide array of insults, the common ones include hypertension, eclampsia and pre-eclampsia, drugs such as cyclosporine and certain infectious causes. Systemic lupus erythematosus (SLE) is one of the rare but established causes of PRES [1]. Central structures, including the basal ganglia, brainstem and bilateral thalami, are rarely involved in PRES and often pose a diagnostic dilemma [2].

## Case presentation

A 39-year-old illiterate female was brought to the emergency room with sudden onset of shortness of breath and loss of consciousness. It was associated with two episodes of generalised tonic-clonic seizures. She had a history of recurrent painless oral ulcers and photosensitivity for the past five years. There was no history of prior seizure activity or any chronic illness, prolonged immobility, recurrent abortions, recent surgery, intake of any medication, substance abuse or hereditary thrombophilic disorder.

On presentation, she had a poor Glasgow Coma Scale (GCS) of 7/15, bilateral coarse crepitations in the chest and reduced capillary oxygen saturation (80% under room air). The patient was afebrile with a regular pulse rate of 110 beats per minute and blood pressure of 118/74 millimeters of mercury. She had a respiratory rate of 20 per minute and her random blood sugar level was normal. There was no neck rigidity. Her electrocardiogram (ECG) was normal except for sinus tachycardia. She was immediately intubated and mechanically ventilated in the intensive care unit (ICU) in view of her deteriorating oxygen saturation.

Radiography of the chest revealed haziness in bilateral lower lung fields suggestive of aspiration pneumonia. Cerebrospinal fluid analysis (protein 18 mg/dL, glucose 62 mg/dL, white blood cells 0/µL and red blood cells 0/µL) along with non-contrast computed tomography (CT) of the head was inconclusive and her routine investigations, including a complete blood count, liver and renal function tests, serum electrolytes, thyroid function tests and fasting blood sugar, were within normal range.

**Figure 1:**
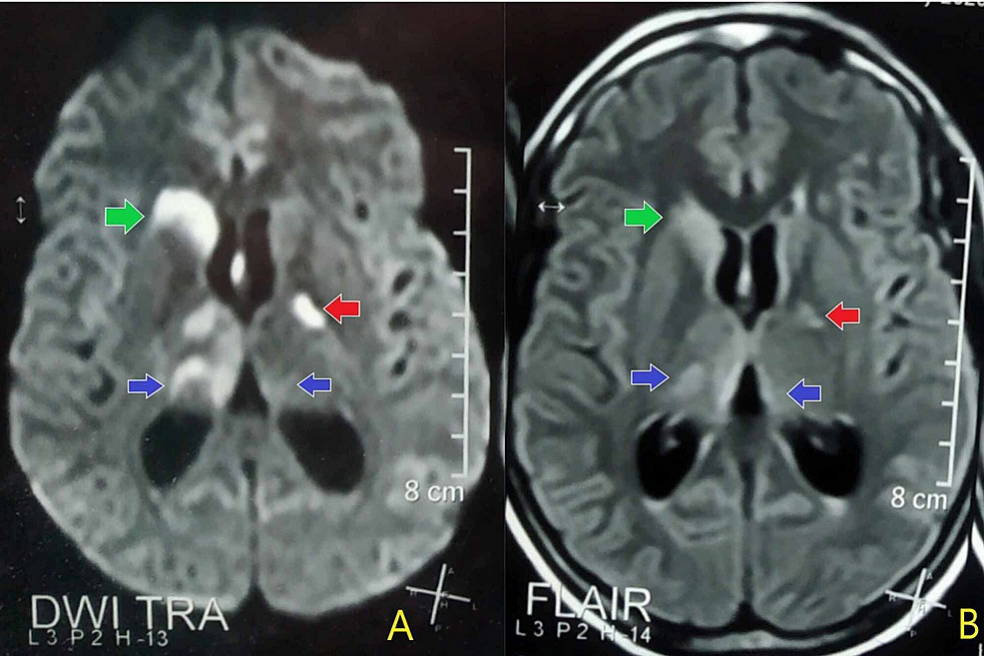
Diffusion restriction in DWI (A) in bilateral thalami (blue arrows), right caudate nucleus (green arrows) and left globus pallidus (red arrows) along with hyperintensity of these regions in (FLAIR) images (B) on MRI brain. DWI: Diffusion-weighted imaging FLAIR: Fluid-attenuated inversion recovery MRI: Magnetic resonance imaging

She was started on intravenous levetiracetam along with ceftriaxone and metronidazole for aspiration pneumonia. COVID-19 was ruled out with reverse transcription-polymerase chain reaction (RT-PCR). Her tracheal culture was sterile after 48 hours of intubation. Her C-reactive protein (CRP) level was 58 mg/L (normal <5 mg/L), antinuclear antibody (ANA) titre was 1:640 (normal <1:160) and anti-double-stranded deoxyribonucleic acid (anti-dsDNA) levels were 147 U/mL (normal <28 U/mL). There was a negligible improvement on this treatment regimen. She was then subjected to magnetic resonance imaging (MRI) of the brain, which indicated areas of signal abnormality in the right caudate nucleus, bilateral thalami and left globus pallidus without the involvement of frontal, parietal and occipital lobes in T2-weighted fluid-attenuated inversion recovery (FLAIR) and diffusion-weighted images. These changes were consistent with central-variant of PRES (Figure 1).

Her urine pregnancy test along with toxicology screen was negative. The patient had worsening of respiratory failure for which levofloxacin was commenced and ceftriaxone was replaced with piperacillin-tazobactum for enhanced microbial coverage. The patient was also started on methylprednisolone (1 gram per day). However, she did not respond to the treatment and unfortunately succumbed to her illness on the fourth day of admission.

## Discussion

PRES is typically a reversible disease which subsides after removal of the offender, but can often result in a dreadful outcome. It comprises vasogenic edema involving various parts of the brain and the symptoms occur in accordance with the involved structures. Two important theories of pathogenesis include raised blood pressure and endothelial dysfunction [3]. A failure of cerebrovascular autoregulation plays a central role in the pathophysiology of PRES and the resultant vasogenic edema [4]. Acute hypertensive crisis is another common cause of PRES [5]. Although SLE is one of the rare causes of PRES, both these mechanisms can be responsible in SLE as these patients can have endothelial dysfunction as well as raised blood pressure secondary to lupus nephritis [6]. PRES is a rare neurological entity in SLE and must be entertained as a differential in patients presenting with neurological signs and symptoms. Close differential possibilities include osmotic demyelination syndrome, alcohol poisoning and central nervous system (CNS) vasculitis.

The symptomatology in patients with PRES is varied, but cognitive disturbances, seizures, headaches and visual field deficits are frequently encountered [7]. MRI brain, especially diffusion-weighted and FLAIR, is the radiological modality of choice for diagnosis of this entity. Complications of PRES stem from increased arterial pressures and may result in intracranial hemorrhage, ischemic cerebral injury and rarely status epilepticus [8]. The central variant of PRES has been documented in patients with SLE, although its incidence remains low [9].

Management of PRES includes removal of the inciting agent and treatment of the underlying cause along with symptomatic care. Although treatment with steroids and cyclophosphamide has been successful in clinical trials, cyclophosphamide is itself a known culprit for PRES and steroids can aggravate hypertension in patients with SLE [10]. These patients, therefore, require intensive monitoring. Being a reversible syndrome, prompt identification of PRES can be potentially lifesaving.

## Conclusions

PRES can occur in patients with SLE and physicians should be adept with patients presenting with the involvement of atypical structures. The central variant of PRES has been documented in patients with SLE, albeit rarely. One needs to be vigilant in promptly diagnosing PRES presenting with the involvement of central structures, in contrast to the typically involved posterior structures. Although its prognosis is dismal, early diagnosis and management can aid in achieving a successful outcome.

## References

[1] Tetsuka S, Ogawa T (2019). Posterior reversible encephalopathy syndrome: a review with emphasis on neuroimaging characteristics. J Neurol Sci.

[2] Bartynski WS, Boardman JF (2007). Distinct imaging patterns and lesion distribution in posterior reversible encephalopathy syndrome. Am J Neuroradiol.

[3] Bartynski WS (2008). Posterior reversible encephalopathy syndrome, part 2: controversies surrounding pathophysiology of vasogenic edema. AJNR Am J Neuroradiol.

[4] Kazmi R, Clark E, Singh V, Falgiani M, Ganti L (2020). Altered and agitated due to hypertension: a case of posterior reversible encephalopathy syndrome. Cureus.

[5] Baig H, Ashraf MH, Ashraf MA, Khan MA, Mohamed Nazeer MN (2020). An unexpected case of posterior reversible encephalopathy syndrome. Cureus.

[6] Hartman EN, Tuna K, Monsour E, Komanduri K, Tarasiuk-Rusek A (2020). Seizure as an initial presentation for posterior reversible encephalopathy syndrome in undiagnosed systemic lupus erythematosus and lupus nephritis: a case report. Cureus.

[7] Lee VH, Wijdicks EF, Manno EM, Rabinstein AA (2008). Clinical spectrum of reversible posterior leukoencephalopathy syndrome. Arch Neurol.

[8] Fischer M, Schmutzhard E (2017). Posterior reversible encephalopathy syndrome. J Neurol.

[9] McKinney AM, Jagadeesan BD, Truwit CL (2013). Central-variant posterior reversible encephalopathy syndrome: brainstem or basal ganglia involvement lacking cortical or subcortical cerebral edema. AJR Am J Roentgenol.

[10] Liu B, Zhang X, Zhang FC, Yao Y, Zhou RZ, Xin MM, Wang LQ (2012). Posterior reversible encephalopathy syndrome could be an underestimated variant of "reversible neurological deficits" in Systemic Lupus Erythematosus. BMC Neurol.

